# A Rare Case of Leukemoid Reaction During Mechanical Circulatory Support in a Patient With Severe Heart Failure: An Autopsy Study

**DOI:** 10.7759/cureus.54603

**Published:** 2024-02-21

**Authors:** Shingo Kunioka, Fumitaka Suzuki, Marino Nagata, Masahiro Tsutsui, Hiroyuki Kamiya

**Affiliations:** 1 Intensive Care Unit/Cardiac Surgery, Asahikawa Medical University, Asahikawa, JPN; 2 Cardiac Surgery, Asahikawa Medical University, Asahikawa, JPN; 3 Pathology, Saitama Medical Center, Kawagoe, JPN

**Keywords:** impella 5.5, extracorporeal membrane oxygenation support, mechanical support, pathological autopsy, medical intensive care unit (micu), heart failure, mechanical circulatory support, leukemoid reaction

## Abstract

The leukemoid reaction (LR) is reported to be caused by severe stress conditions such as infection, malignancies, intoxication, severe hemorrhage, or acute hemolysis; this condition is attributed to a very severe prognosis. Some reports have suggested that the LR was associated with a systemic stress response. A 36-year-old man who required mechanical circulatory support (MCS), including veno-arterial extracorporeal membrane oxygenation and Impella 5.5 due to severe heart failure, was transferred to our hospital. He showed a markedly elevated WBC count and died of multiple organ failure. The autopsy revealed the possibility that leukocytosis might have been due to an LR; however, the cause of the cardiac failure was unknown. To the best of our knowledge, this study is the first to report a rare case of LR in a patient with severe heart failure requiring MCS.

## Introduction

A leukemoid reaction (LR) is defined as a condition characterized by a leukocyte count greater than 35,000-50,000 cells/μL [1-3]. Some causes of LR have been reported in previous reports, which indicated that LR can be caused by severe infection, malignancies, intoxication, severe hemorrhage, or acute hemolysis [3]; LR can lead to a very severe prognosis [4-6]. Furthermore, only a few reports suggest that it also occurs under stressful conditions, resulting in multi-organ failure [7-10]. Although severe heart failure requiring mechanical circulatory support (MCS) is a severely stressful condition, there are no reports suggesting an association between LR and severe heart failure. Herein, we report the first case suspected of showing LR during MCS, including extracorporeal membrane oxygenation and Impella 5.5, where an autopsy was performed for detailed evaluation.

## Case presentation

**Figure 1:**
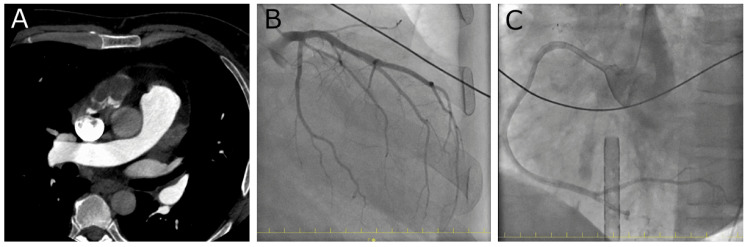
Enhanced computed tomography and coronary angiography findings. (A) Computed tomography findings showed no evidence of pulmonary thromboembolism, although a right atrial thrombus was present. (B, C) Coronary angiography revealed no signs of coronary artery disease.

A 36-year-old man with no notable medical history was admitted to a local hospital because of severe dyspnea and a fever lasting for a week. He also complained of swelling and pain in the left lower extremity. Enhanced computed tomography revealed a deep vein thrombus in the left lower extremity. He experienced tachycardia with a heart rate of over 200 bpm and a sudden progression of circulatory deterioration. Bedside echocardiography revealed a severely reduced left ventricular ejection fraction (10%); veno-arterial extracorporeal membrane oxygenation (VA-ECMO) was immediately introduced from femoral vessels. Emergency coronary artery angiography was performed, revealing no signs of coronary artery disease (Figure 1).

Laboratory examinations indicated an increased WBC count of 15,600/µL (normal range: 3,500-9,100/µL, %neut 72.4%, %lymph 18.8%, %mono 8.2%, %baso 0.3%), an elevated RBC count of 578 × 10^4^/µL (normal range: 400-550 × 10^4^/µL), and C-reactive protein (CRP) levels of 1.5 mg/dL (normal range: <0.10 mg/dL). Creatine phosphokinase was not elevated. The patient was transferred to our institution the following day for advanced heart failure care, including long-term mechanical circulatory support (MCS). We performed Impella 5.5 insertion via the right axillary artery. By the time he arrived at our hospital, his liver and renal function were already impaired (serum albumin (Alb) 1.3 g/dL, serum bilirubin (Bil) 4.4 mg/dL, serum cholinesterase (Che) 75 IU/L, prothrombin time (PT) 86.9 sec, estimated glomerular filtration rate (eGFR) 26.0 mL/min). The patient did not develop a fever during the course of treatment, blood cultures were repeatedly negative, and imaging studies showed no evidence of pneumonia. Despite the absence of signs of infection, blood examination showed a rapid increase in WBC counts, with the peak level at 49,000 cells/μL. Despite continuous MCS, he died of multiple organ failure (MOF, Bil 32.8 mg/dL, eGFR 14.7 mL/min), 18 days after admission.

**Figure 2:**
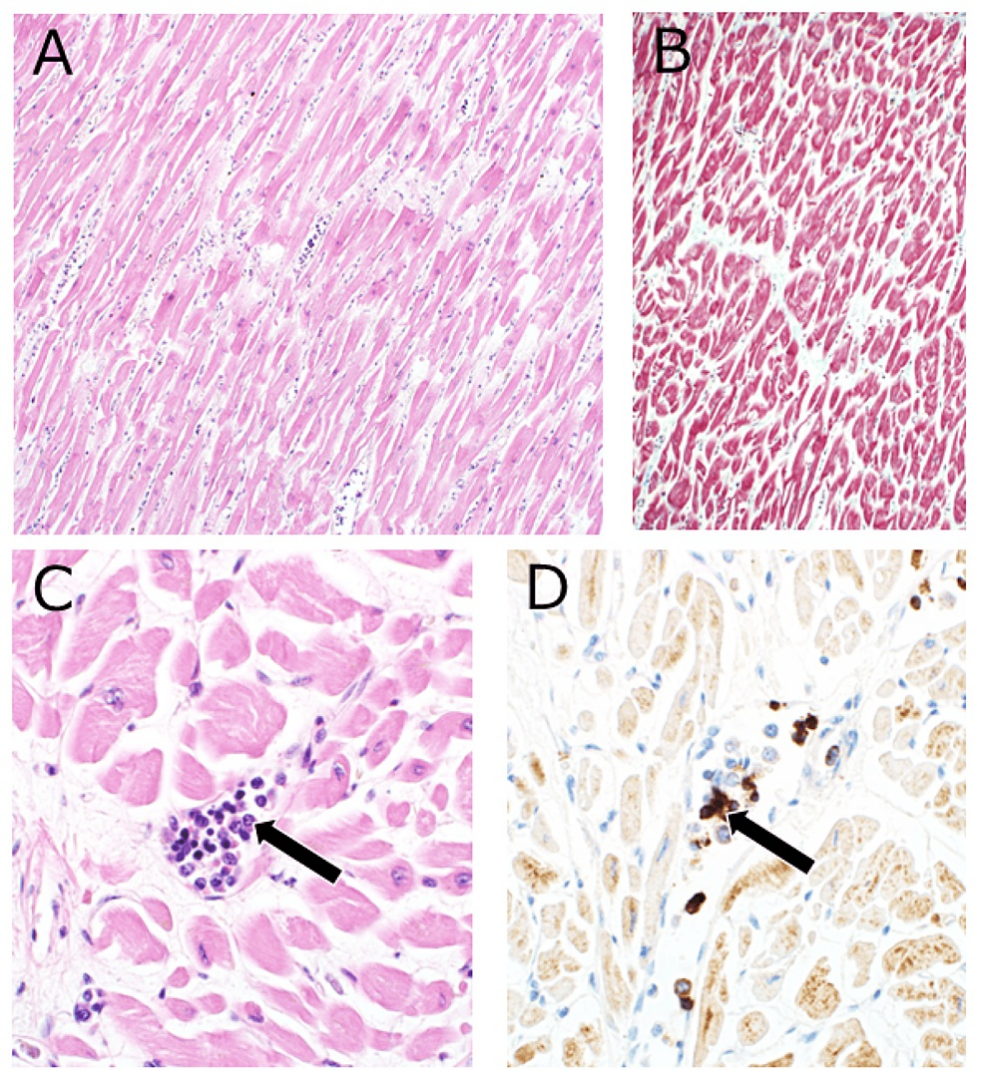
Images of the autopsy examination – the heart muscle. (A) Cardiomyocytes showed mild hypertrophy and fibrosis between cardiomyocytes, with no obvious convoluted arrangement, necrosis, or inflammatory cell infiltration. (B) Masson's trichrome staining showed little staining, suggesting that fibrosis had not significantly developed. (C) The conspicuous presence of cells in the capillaries that were larger and more atypical than normal blood cells (arrow). (D) CD71-positive erythroblasts, indicating the appearance of erythroblasts in the peripheral blood (arrow).

His family agreed to an autopsy; the autopsy was performed to determine the cause of severe heart failure and leukocytosis, leading to his death. As for the cause of severe heart failure, myocardial findings did not show myofibrillar disarray; thus, the cause remained unknown (Figure 2A). However, a large number of erythroblasts appeared in the capillaries (Figure 2B). The conspicuous presence of cells in the capillaries that were larger and more atypical than normal blood cells suggested that these cells were CD71-positive erythroblasts, appearing in the peripheral blood (Figures 2C-2D).

**Figure 3:**
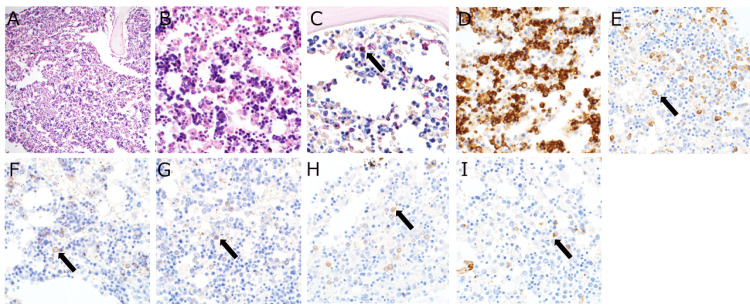
Images of the autopsy examination - the bone marrow. (A, B) Bone marrow findings showing hyperplastic bone marrow with abnormal proliferation of erythroblastic cells. (C) Naphthol AS-D chloroacetate esterase staining showing a decrease in myelomonocytic cells with red staining of the cytoplasm (arrow). (D) Erythroblastic cells positive for CD71 accounted for 70% of all nucleated cells (arrow). (E) Myeloid cells positive for myeloperoxidase represent less than 20% (arrow). (F, G) Immature erythroblasts positive for E-cadherin (E, arrow) and some p53-positive cells (G, arrow) were observed. (H, I) Scattered large cells, positive for c-kit (H, arrow) and CD34 (I, arrow), were observed, comprising 5% of all nucleated cells, suggesting an increase in blasts.

As for the cause of extreme leukocytosis, bone marrow findings showed hyperplastic bone marrow with abnormal proliferation of erythroblastic cells, which accounted for more than 70% of all nucleated cells (Figures 3A-3I). Naphthol AS-D chloroacetate esterase staining showed a decrease in myelomonocytic cells with red staining of the cytoplasm, indicating that the other cells were predominantly erythroblastic (Figure 3C). Erythroblastic cells positive for CD71 accounted for 70% of all nucleated cells, while myeloid cells positive for myeloperoxidase were less than 20% (Figures 3D, 3E). Immature erythroblasts positive for E-cadherin and some p53-positive cells were also observed (Figures 3F, 3G). There were scattered large cells positive for c-kit and CD34, comprising about 5% of all nucleated cells, suggesting an increase in blasts (Figures 3H, 3I).

A hematological malignancy disease, erythroleukemia, was suspected; however, the diagnosis could not be made because of the absence of a blood smear; thus, LR was suspected comprehensively.

## Discussion

LR results in poor prognosis, and no effective LR management has been established to date [4-6]. This case report highlights three practical suggestions. Firstly, to the best of our knowledge, this is the first report to describe a patient requiring VA-ECMO and Impella support who developed LR and died of MOF. Secondly, rapid progression of extreme leukocytosis in patients with severe heart failure requiring MCS should be managed considering that they might have non-infectious leukemia, such as leukemia analogs, as well as infectious diseases. Lastly, this is a valuable case where a detailed evaluation of heart failure and hyperleukocytosis was performed at autopsy.

The definition of LR varies; however, it is generally defined as a condition in which the WBC count increases to 35,000-50,000 cells/μL [1-3]. Our patient exhibited an elevated WBC count of up to 49,000 cells/μL, suggesting he might have developed LR. LR is associated with a high fatality rate and poor prognosis; this patient also died of MOF. This report suggests that cases of severe heart failure requiring MCS can develop LR due to MOF; hence, the possibility that increased WBC counts may be due to non-infectious diseases should be considered. Moreover, Kyne L et al. reported an association between the development of congestive heart failure and LR [9], though their patient group had acute myocardial infarction. The present case, when considered in light of the autopsy findings, suggested that this patient might have developed cardiomyopathy, including tachycardia-induced cardiomyopathy, indicating that not only acute myocardial infarction but also heart failure due to such cardiomyopathy may lead to LR.

Autopsies are essential for understanding the pathology, and in this case, there were several interesting findings, especially myocardial and bone marrow findings. Myocardial pathological examinations revealed no typical findings of cardiomyopathy, such as myofibrillar disarray, cardiac sarcoidosis, or ischemic cardiomyopathy, but only nonspecific findings. Based on these findings, the patient may have developed severe acute heart failure due to tachycardia-induced cardiomyopathy, and the elevated white blood cell count was presumed to be due to stress-induced LR from acute heart failure. Also, the intracapillary space was filled with many erythrocytes (Figure 2). Moreover, bone marrow findings showed hyperplastic bone marrow with abnormal proliferation of erythroblastic cells (Figure 3). Considering these findings, the patient was suspected of having had the possibility of developing leukemia, such as erythroleukemia; however, clinically, it was considered more likely to be leukocytosis due to LR, associated with a stress response to acute heart failure, because smears were required to diagnose erythroleukemia.

## Conclusions

In this case, the autopsy examination revealed that the elevated WBC counts during the treatment of severe heart failure with MCS were actually due to LR rather than infection, which may be of clinical value. LR in severe heart failure, especially in those on MCS, has not been previously reported. It is essential that increased WBC counts in patients with severe heart failure are treated with the possibility of LR in mind, rather than assuming that they are due to infection.
